# Spinal cord compression: an unusual presentation of hepatocellular carcinoma

**DOI:** 10.11604/pamj.2014.19.363.4323

**Published:** 2014-12-09

**Authors:** Hileni Taleni Nangolo, Larrea Roberto, Innocent Lule Segamwenge, Andreas Voigt, Fredrick Kidaaga

**Affiliations:** 1 Department of Internal Medicine, Intermediate Hospital Oshakati, Oshakati, Namibia; 2 Department Orthopedic Surgery, Intermediate Hospital Oshakati, Oshakati, Namibia; 3 Namibian Institute of Pathology, Khomas, Namibia

**Keywords:** Hepatocellular carcinoma, spinal cord compression, spine metastases

## Abstract

Hepatocellular carcinoma is the 5^th^ most common cancer in men and the 2^nd^ common cause of death from cancer worldwide. The tumour commonly metastasizes to the lungs, regional lymph nodes and bone. Spinal cord compression secondary to metastatic disease as a first presentation is uncommon. We describe a patient who presented with paraplegia as a first presentation of hepatocellular carcinoma. 46 year old Namibian man presented with progressive leg weakness that was associated with a dull back ache and inability to pass urine and stool. He had no history of trauma nor did he have chronic cough, night sweats or fevers. He has been treated several times for alcohol dependence. On examination he was wasted, power 0/5 in both lower limbs and a sensory level at T12. He also had a non-tender hepatomegaly with Alpha-fetoprotein of 2000. The Chest X-ray and Chest CT showed nodular opacities indicating metastatic disease and the X-ray and CT of the thoracic spine showed osteolytic lesion with destruction of the pedicle of L1. Liver and spinal biopsy confirmed the hepatocellular carcinoma. The extra hepatic manifestations of HCC are diverse and Spinal cord metastasis is of pertinent clinical importance and should thus be greatly considered.

## Introduction

Hepatocellular carcinoma (HCC) is a common tumour in sub-Saharan Africa and a common cause of death in the continent [1]. This big burden of the tumour is largely driven by the high prevalence of Hepatitis B virus infection (HepB) in Africa. The tumour commonly presents as right upper quadrant abdominal pain with a mass in association with other features of hepatic decompensation. The tumour commonly metastasizes to the lungs, regional lymph nodes and bone [2, 3]. Initial presentation with spinal cord presentation is rare to our knowledge only a few case reports have described such a presentation. We describe a patient who presented with spinal cord compression as the first manifestation of hepatocellular carcinoma.

## Patient and observation

A 46 year old Namibian male was referred to our hospital from a district Hospital with complaints of legs weakness for 3 weeks. The patient was in his usual state of health until 3 weeks prior, when he developed progressive leg weakness associated with dull back pain localized to thoracic area. He also noticed inability to pass urine and stool. There was no history of trauma to the back preceding this presentation. There were no complaints related to other body systems except general malaise and weight loss. His past medical history was significant for being on treatment for schizophrenia which was well controlled on haloperidol. In addition he had been treated several times for alcohol dependence. On physical examination we found a middle aged man, sick looking and markedly wasted. He was apyrexial with finger clubbing and below knee bilateral pitting edema. His Vital signs were normal. He had reduced muscle bulk in all limbs reflecting generalized wasting. The tone in the lower limbs was reduced, with power of 0/5 bilaterally and absent knee and ankle reflexes. The Babinski sign was present and he had a sensory level at T12. The upper limbs were normal with power of 5/5. The abdomen was mildly distended, with a firm non tender hepatomegaly 4cm below costal margin. There was no splenomegaly and the kidneys were not palpable. The lungs were clear on auscultation.

**Figure 1:**
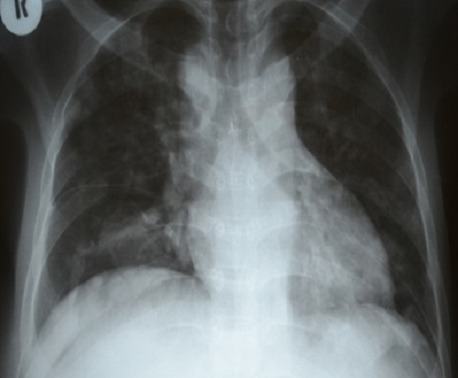
Chest x-ray showing nodular opacities in both lung fields

**Figure 2:**
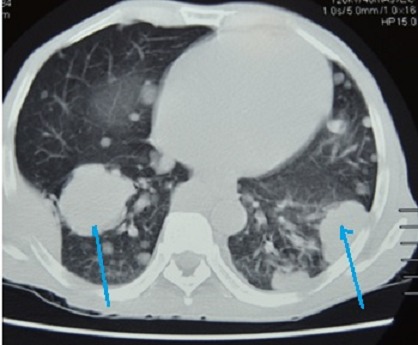
Chest CT Scan of showing lung masses (blue arrows) and multiple nodules consistent with metastatic lung disease from Hepatocellcarcinoma

**Figure 3:**
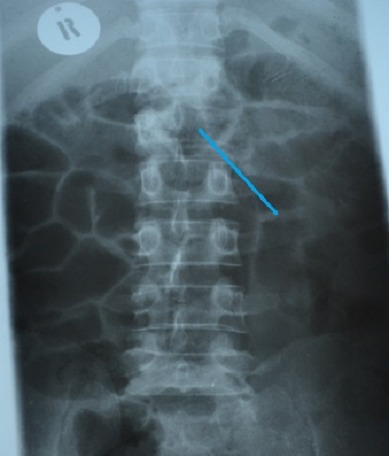
Thoracic spine x-ray showing Osteolytic Left L1 lesion with missing pedicle (blue arrow)

**Figure 4:**
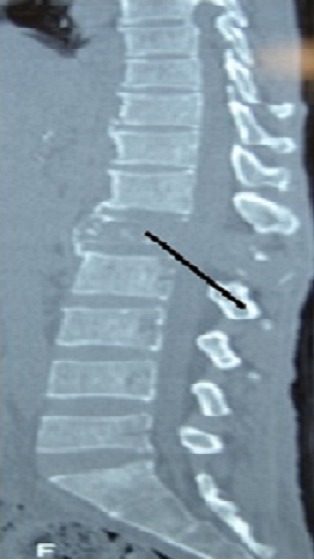
CT scan showing L1 osteolytic vertebra body lesions (black arrow) with a soft tissue swelling and L4 osteopenia sparing intervertebral disk spaces

The patient liver function tests were consistent alcoholic hepatitis with a ratio of aspartate transaminase (AST) 180 IU/l to alanine transaminase (ALT) 70 IU/l greater than 2:1. The alkaline phosphatase (ALP) and Gamma-glutamyltransferase (GGT) were high 269 IU/l and 223 IU/l respectively. He had hypoalbuminemia of 25g/l and the serology for hepatitis B was consistent with chronic hepatitis B infection and Hepatitis C serology was unreactive. His Alphafeto protein was elevated 2000 ng/ml and the International normalized ratio (INR) was normal at 0.98. The abdominal ultrasound revealed the liver to be enlarged with multiple hyperechoic masses biggest measuring 4x4 cm in diameter and ascites. The Chest X Ray and Chest Computed Tomography (CT) Scan showed bilateral nodular lesions in keeping with pulmonary metastatic disease. (Figure 1, Figure 2). Spinal X Ray and CT scan showed lumbar 1 (L1) osteolytic vertebra body lesions with left pedicle destruction and L4 osteopenia without anterior wedging, kyphosis or destruction of the adjacent intervertebral disk spaces (Figure 3, Figure 4).

**Figure 5:**
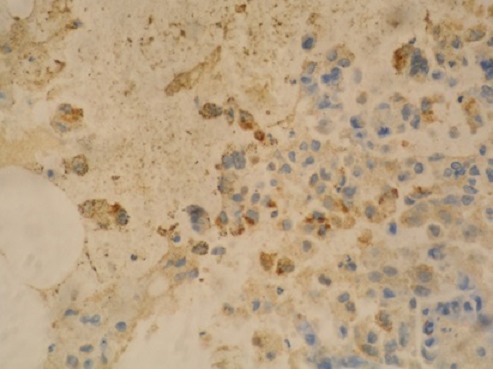
Histological illustration of an AFP positively stained bone mass

**Figure 6:**
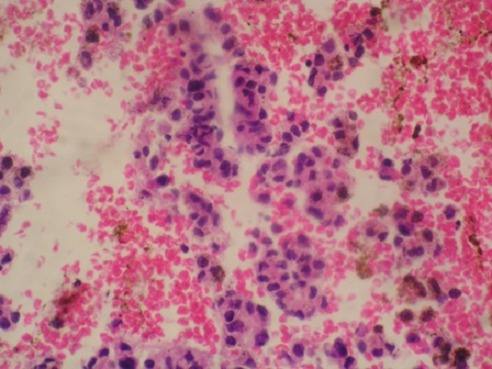
H&E Bone mass

A liver biopsy was done which showed features of liver cirrhosis and hepatocellular carcinoma. Computed tomography guided biopsy and histology of the paravertebral mass showed features consistent with a metastatic hepatocellular carcinoma and the abnormal cells were positive to immunostains for Hep par 1 (Figure 5, Figure 6). The patient was not offered palliative radiotherapy for his spine metastases because the spinal cord compression had lasted over 3 weeks. He was offered tramadol and was discharged on morphine and lactulose for his pain control and supportive nursing care. He expired after one month hospital stay.

## Discussion

Hepatocellular carcinoma is the 5^th^ most common cancer in men and the second most common cause of death from cancer worldwide [4]. It is largely a problem of less developed regions accounting for 83% of new cases of hepatocellular cancer worldwide. This high burden of the cancer in largely driven by the high burden of hepatitis B virus infection in these regions. Other risk factors for HCC include hepatitis C virus infection, aflatoxin, alcoholic liver disease and rarer diseases like Wilson's disease, haemochromotosis and alpha 1 antitrypsin deficiency. Our patient had two major risk factors associated with development of HCC were present; the history of Hepatitis B infection and the alcohol induced liver damage.

Most patients with HCC present with right upper quadrant abdominal pain, solitary or diffuse abdominal mass and hepatomegaly [5]. However, patients may have initial symptoms that are related exclusively to extrahepatic metastases [5]. Extrahepatic metastases are associated with advanced intrahepatic tumours, vessel invasion and reduced 1 year survival. The lungs are the most frequent metastatic site for HCC followed by lymph nodes, bone and adrenal glands [3]. Among patients with bone metastases the vertebrae is the most common site followed by the pelvis, ribs and skull respectively [6]. Our patient did not have any symptoms related to the primary tumor. Rather, he had an extrahepatic presentation; paraplegia as consequence of spinal cord compression secondary to HCC metastases. Metastatic presentation with spinal cord compression is unusual, however there is an expanding body of evidence in the medical literature reporting this type of presentation of HCC metastases [7–10]. Our patient had jaundice, bilateral leg oedema, finger clubbing and hepatomegaly. In addition the abdominal ultrasound showed an enlarged liver with multiple masses and alpha feto-protein level of greater than 2000. These were suggestive signs to a possibility of a neoplastic process. Metastases were also confirmed in the lungs on plain chest x-ray and computed tomography (Figure 1, Figure 2). Biopsy of the liver and vertebrae confirmed metastatic hepatocellular carcinoma as the cause of spinal cord compression.

Spinal cord compression in sub-Saharan Africa is commonly due to Tuberculosis (TB) of the spine and prostate Carcinoma of older men. Our patient presented with a mass at the level of the 10^th^ to 11^th^ thoracic vertebrae, weight loss and paraplegia. Although the patient did not have other constitutional symptoms of fever and night sweats, these features were initially suggestive of a diagnosis of tuberculosis of the spine. The paravertebral mass was similar to a gibbus seen in patients with spinal TB. However, spinal plain X-rays and computed tomography scan of the spine showed osteolytic destructive lesions involving the first lumber vertebra but affecting posterior vertebral body and left pedicle, sparing intervertebral disk space (Figure 3). TB of the spine is more common in children and young adults. In TB of the spine characteristically there is destruction of the intervertebral disk space and the adjacent vertebral bodies, collapse of the spinal elements, and anterior wedging. These changes lead to kyphosis and gibbus formation in the thoracolumbar region of vertebral column most frequently affecting the mid-thoracic vertebrae. Since both tuberculosis and hepatocellular carcinoma are common in the less developed regions of the world, a broader differential diagnosis is required when evaluating patients with paraplegia in this part of the world.

## Conclusion

It is worth stressing that HCC should be included in the differential diagnosis of metastatic extradural spinal cord compression, because it may be the initial manifestation, with or without overt signs of liver disease as occurred in our patient.
